# Effect of Branding and Familiarity of Soy Sauces on Valence and Arousal as Determined by Facial Expressions, Physiological Measures, Emojis, and Ratings

**DOI:** 10.3389/fnrgo.2021.651682

**Published:** 2021-05-13

**Authors:** Rene A. de Wijk, Shota Ushiama, Meeke J. Ummels, Patrick H. Zimmerman, Daisuke Kaneko, Monique H. Vingerhoeds

**Affiliations:** ^1^Wageningen Food and Biobased Research, Wageningen University and Research, Wageningen, Netherlands; ^2^Kikkoman Europe R&D Laboratory B.V., Wageningen, Netherlands; ^3^Noldus Information Technology, Wageningen, Netherlands

**Keywords:** skin conductance, heart rate, ANS responses, branding, product usage

## Abstract

Food experiences can be summarized along two main dimensions: valence and arousal, which can be measured explicitly with subjective ratings or implicitly with physiological and behavioral measures. Food experiences are not only driven by the food's intrinsic properties, such as its taste, texture, and aroma, but also by extrinsic properties such as brand information and the consumers' previous experiences with the foods. In this study, valence and arousal to intrinsic and extrinsic properties of soy sauce were measured in consumers that varied in their previous experience with soy sauce, using a combination of explicit (scores and emojis), implicit (heart rate and skin conductance), and behavioral measures (facial expressions). Forty participants, high- and low-frequency users, were presented with samples of rice and three commercial soy sauces without and with brand information that either matched or non-matched the taste of the soy sauce. In general, skin conductance and facial expressions showed relatively low arousal during exposure to the brand name and again lowest arousal during tasting. Heart rate was lowest during exposure to the brand name and increased during tasting probably resulting from the motor activity during chewing. Furthermore, the results showed that explicit liking and arousal scores were primarily affected by the taste of the specific soy sauce and by the participants' previous experience with soy sauces. These scores were not affected by branding information. In contrast, facial expressions, skin conductance, and heart rate were primarily affected by (1) the participants' level of experience with soy sauce, (2) whether or not branding information was provided, and (3) whether or not the branding information matched with the taste. In conclusion, this study suggests that liking scores may be most sensitive to the food's intrinsic taste properties, whereas implicit measures and facial expressions may be most sensitive to extrinsic properties such as brand information. All measures were affected by the consumers' previous food experiences.

## Introduction

Food perception typically starts before the food is placed in the mouth. Before the food is consumed, consumers typically smell the food and see the food, often with the food package with brand name. Consequently, prior to tasting, consumers will already have expectations regarding the food's taste and flavor based on visual, smell, tactile, and sometimes even auditory cues. These expectations affect the decision of the consumer, whether the food is to be consumed. Furthermore, when the consumer decides to consume the food, expectations on food affect the taste experience as well.

Numerous studies have focused especially on visual cues. They have demonstrated the effects of branding, names and sensory descriptors, and health and ingredient labels on taste experiences [see Piqueras Fiszman and Spence (2015) and Skaczkowski et al. (2016) for excellent reviews of these effects]. Models such as the assimilation and contrast model (Schifferstein et al., 1999; Piqueras Fiszman and Spence, 2015) describe various ways of how expectations interact with actual experiences, depending on their overlap and discrepancies. In the assimilation model, the discrepancy is relatively small, and experiences are adjusted to the expectations. In contrast, when the discrepancy is relatively large, the differences between expectations and experiences are amplified. As a result, ratings for the experience will shift in the opposite direction of the expectations.

Even though the effects of expectations on food experiences are well-documented, the results provide relatively little insight into the underlying mechanism. This mechanism is probably complex due to the multiple factors as well as their interactions. Factors include arousal, valence, novelty/familiarity, attention, and relevance. These factors have been the cornerstone of various theories of emotion (e.g., Moors et al., 2013; Coppin and Sanders, 2016). The so-called appraisal theories, such as the component process model (e.g., Coppin and Sanders, 2016), focus on the highly dynamic nature of emotional responses. Appraisal theories assume that different stages of the temporal development of responses reflect different appraisals of stimuli, such as the stimulus' novelty, relevance or pleasantness. According to the component process model, an event's significance is evaluated on several criteria in a fixed temporal sequence. These appraisal criteria are: (1) Relevance (how relevant is this event for me), (2) Implication (what are the consequences of this event and how does it affect my well-being and goals), (3) Coping (how well can I adjust to these consequences), and (4) Normative significance (what does this event mean with respect to my self-concept and to social norms and values). Each criterion is associated with specific appraisals. For example, when a stimulus such as a food is encountered, the first and fastest appraisals concern the relevance of the stimulus for the consumer. These relevance appraisals include appraisals of the stimulus' novelty, its predictability (e.g., did this food meet expectations?), its intrinsic pleasantness, and the relevance of the stimulus for the perceiver's goals. Each of these appraisals triggers specific physiological responses (action tendencies, motor expressions, and subjective feeling responses), each with its own specific function. Action tendency refers for example to whether or not the stimulus may be a threat that requires avoidance, whereas motor expressions serve to communicate this possible threat to others *via* facial expressions. The relevance appraisal is followed by appraisal of the possible implications of the stimulus for the perceiver. These appraisals are different from the previous relevance appraisals, and will trigger a different set of reactions, and so on. Understanding the mechanism underlying expectations and experiences will, therefore, facilitate the understanding of emotions, which are an important driver of food choice.

Most studies used subjective ratings and questionnaires, so-called explicit measures, to assess the effects of expectations on taste experiences, i.e., these studies rely on introspection while consumers may not even be aware of the way expectations affect their experiences (e.g., Yeomans et al., 2008). Additionally, virtually all studies measured the effect of expectation indirectly by measuring its effect on subsequent taste experiences, i.e., only very few studies try to measure the expectations themselves, except for Thomson, who uses a specially developed technique (Thomson, 2010).

On the other hand, implicit measures of autonomic nervous system (ANS) responses, such as heart rate and skin conductance, and their behavioral correlates, such as facial expressions, may offer a promising alternative. In fact, these measurements (1) do not rely on introspection and offer insight into the subconscious as well as conscious processes and (2) can be measured continuously during expectations and experiences (e.g., De Wijk and Boesveldt, 2016). Mandler's schema incongruity theory combines ANS responses and the assimilation/contrast model mentioned above. According to Mandler, the size of the ANS response varies directly with the discrepancy between expectation and experiences (Mandler, 1982).

In food science, implicit measures have been primarily used to complement explicit measures during the tasting. The results of various studies suggested little added value of implicit measures over explicit measures (Danner et al., 2014; De Wijk et al., 2014; Mojet et al., 2015; Samant et al., 2017; Kaneko et al., 2019a; De Wijk and Noldus, 2021). Typically, ANS responses varied little between foods, and/or ANS responses were difficult to relate to explicit responses. Some of these results may be related to artifacts in ANS responses caused by interference from motor activity while chewing or drinking. This interference may blur any effects of factors such as novelty and valence. Such interference should not occur when expectations are formed during a visual inspection of brand information, such as brand names and/or packages. So far, this application of ANS measurements has been rarely used in food studies (e.g., Verastegui-Tena et al., 2019a). In non-food studies on expectations, ANS measurements typically include skin conductance and heart rate. Skin conductance is typically seen as a reliable marker for arousal (e.g., Boucsein, 1992). Novelty is often also associated with increased skin conductance/arousal, often combined with a short deceleration in heart rate [also referred to as orienting response, see Bradley (2009), Verastegui-Tena et al. (2019a), and Verastegui Tena et al. (2019b)]. Attention and anticipation are associated with a more prolonged deceleration in heart rate (Poli et al., 2007). Facial expressions, the expressive component of emotions, relates to factors such as arousal, novelty, and valence (e.g., Ekman, 1999). Studies, especially in the non-food domain, have demonstrated that high arousal is typically associated with emotions of anger, disgust, fear, and happiness. Novelty is associated with emotions of surprise and interest, familiarity with neutral, or even boredom (e.g., Coppin and Sanders, 2016). Valence may be seen as a combination of emotions of (lack of) happiness and (lack of) negative emotions such as disgust, anger, and fear. Combined, ANS responses and facial expressions may be well-suited to explore the roles of arousal, novelty/familiarity, valence, and attention in expectations and experiences of foods.

This study focuses on two main dimensions of experiences: valence and arousal. Valence was measured subjectively with liking scores, physiologically with heart rate, and expressively with facial expressions related to valence. Arousal was measured subjectively with arousal scores, physiologically with skin conductance, and expressively with facial expressions related to arousal.

This study explored the effects of branding and familiarity with ANS measures, facial expressions, and explicit ratings for products from a single product category, namely soy sauces. Soy sauce is a condiment with a distinctive flavor that is familiar to most Asian consumers and relatively unfamiliar to many Western consumers and associated with a limited range of foods, such as sushi. Previously, soy sauces were mainly offered in speciality shops targeted at specific consumers. However, nowadays, soy sauces are more and more offered in regular supermarkets to the mainstream consumer in a large variety, and applications of soy sauces are no longer limited to sushi but expand to daily meals, such as stir-fried foods. Despite its growing popularity, soy sauce is still consumed regularly by a relatively small group of consumers, while most consumers either never consume soy sauce or very infrequently (Fenko et al., 2015). Recently, the frequency of use of soy sauce was related to factors such as food neophobia and preferences for soy sauces in general as well as preferences for specific brands of soy sauce (Ushiama et al., 2021).

Expectations regarding soy sauces were varied in this study by (1) including participants with various degrees of familiarity with soy sauces, (2) including soy sauces that are either available or not available in Dutch supermarkets, and (3) presenting the soy sauces without brand information and with matching- and non-matching branding information. The continuous nature of the implicit measures allowed us to measure not only responses to the taste of soy sauces but also responses to the preceding brand information. Responses to the taste stimulus reflect the combined effect of the interaction between expectations and actual tasting, whereas the responses to the preceding phases reflect the effect of only the expectations.

We hypothesize that (1) expectations will vary with the frequency of soy sauce use, (2) different brand information (product) will produce different expectations, and (3) expectations affect responses to the brand name and to the taste stimulus. Strong expectations based on the brand information are associated with (1) strong responses during the viewing of the brand information, (2) weak responses during the tasting of the matching taste due to confirmation, (3) strong responses during the tasting of a non-matching taste due to disconfirmation. Expectations increase with (1) usage of soy sauce and (2) familiarity with the brand.

## Methods

### Participants

Forty healthy participants (30 females and 10 males aged between 20 and 59 yrs. with an average age of 43 yrs.) were recruited from the consumer database of Food, Health and Consumer Research (Wageningen Food and Biobased Research) after screening with an online survey (EyeQuestion Software, Logic8 B.V., the Netherlands). The data base consists of 2,945 persons (1,015 males and 1,930 females all living in or near Wageningen and aged between 19 and 95 yrs.). Exclusion criteria were allergies or intolerance to wheat, gluten, soybean, or rice allergy. All participants provided written consent to participate in the experiment. In the recruitment questionnaire, participants were asked to indicate the frequency of their soy sauce use using an 8-point scale (more than three times a week, once of twice per week, once per month, once per 3-4 month, once or twice per year, less than once a year, never used, I don't know if I use soy sauce) and daily use soy sauce brand. The participants were assigned to one of two groups depending on their frequencies of use. Twenty-two participants, 17 females and five males, who used soy sauce either never or maximum once per month were assigned to the *Low-frequency user* group. Eighteen participants, 15 females and three males, who used soy sauce at least once per week were assigned to the *High-frequency user* group.

The participants received financial compensation of 10 euro after finalizing the study. The study was approved by the Social Ethical Committee of the Wageningen University and Research Center.

### Materials

#### Soy Sauces

Three soy sauces were selected based on the similarity of ingredients and availability in the Netherlands. Based on a previous study (Ushiama et al., 2021), two familiar brands of soy sauces [Kikkoman (Kikkoman Foods Europe B.V., Sappemeer, The Netherlands) and Inproba (Inproba B.V., Baarn, The Netherlands)] were selected that shared the typical savory salty taste of soy sauce and one unfamiliar brand of soy sauce with a clearly different taste [Maekrua Gold Label® Soya Sauce Formula 1 (Mae Krua Sri Ruen Company Limited, Bangkok Thailand)]. In order to study the effect of brand familiarity, the familiar Inproba soy sauce was presented in an unfamiliar bottle (Wan Ja Shan from Taiwan). Samples of the soy sauces (1 ml, either with or without branding information) were mixed together with 16 g of shaped Sticky-rice (Yumenishiki, JFC Deutschland GmBH, Düsseldorf, Germany) in a styrofoam cup and presented at 60°C. Although the previous study showed sensory characteristics vary by carrier (Cherdchu and Chambers, 2014), white rice with soy sauce was selected because it is a relatively common way to use soy sauce in the Netherlands. Rice had been cooked in a commercial rice cooker and kept warm in the incubator and portioned before each session.

### Procedure

Participants participated in one 1-h session, which started with an explanation of the procedure, followed by the attachment of the skin conductance and heart rate sensors on the fingers and palm of the non-dominant hand, and by two practice trials. Participants were seated in front of a laptop and a webcam (Microsoft LifeCam) which recorded the participant's face. Instructions and images of the unbranded or branded soy sauces were shown on the laptop screen using E-Prime software (E-Prime, Science Plus Group, The Netherlands). Participants were instructed that their perception of soy sauces was studied, and that to avoid unnecessary movements that may interfere with the measurements, small samples of rice with soy sauce would be put in their mouth by the experimenter. The sample should be chewed normally, and the participant was instructed to signal the swallow with a hand signal after which scores were entered by a mouse click.

**Figure 1:**
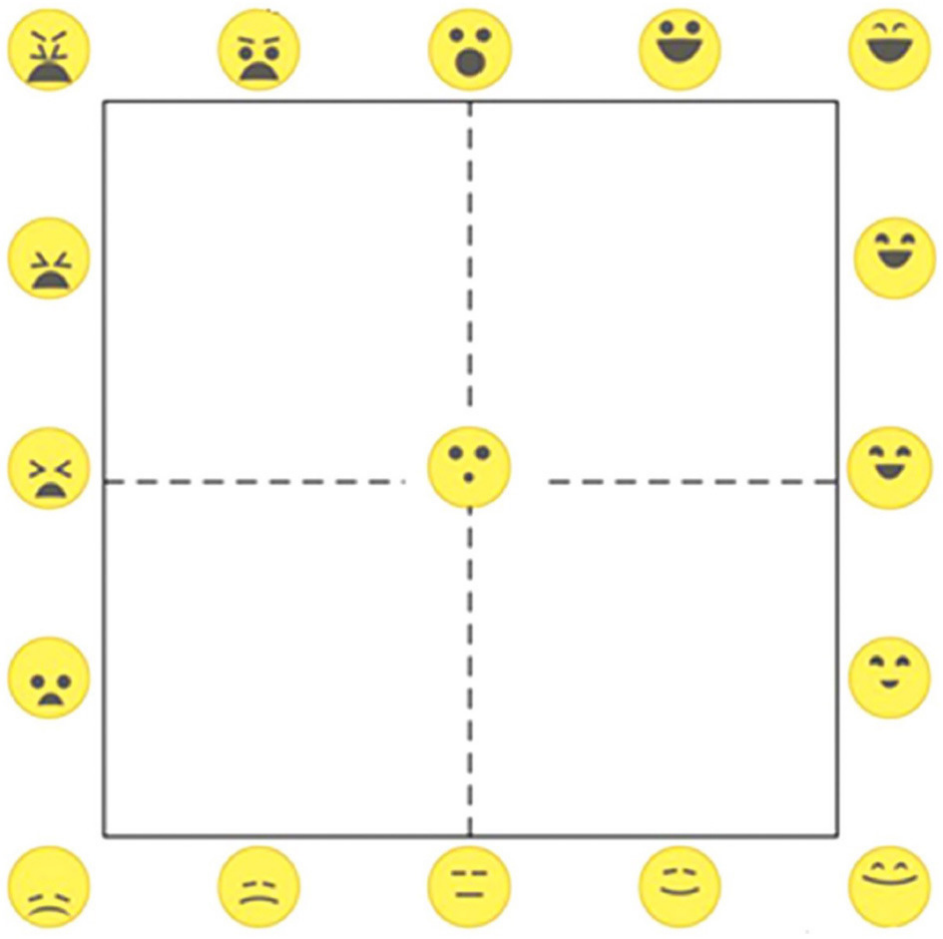
The EmojiGrid used in the study to measure valence and arousal.

**Scheme 1:**
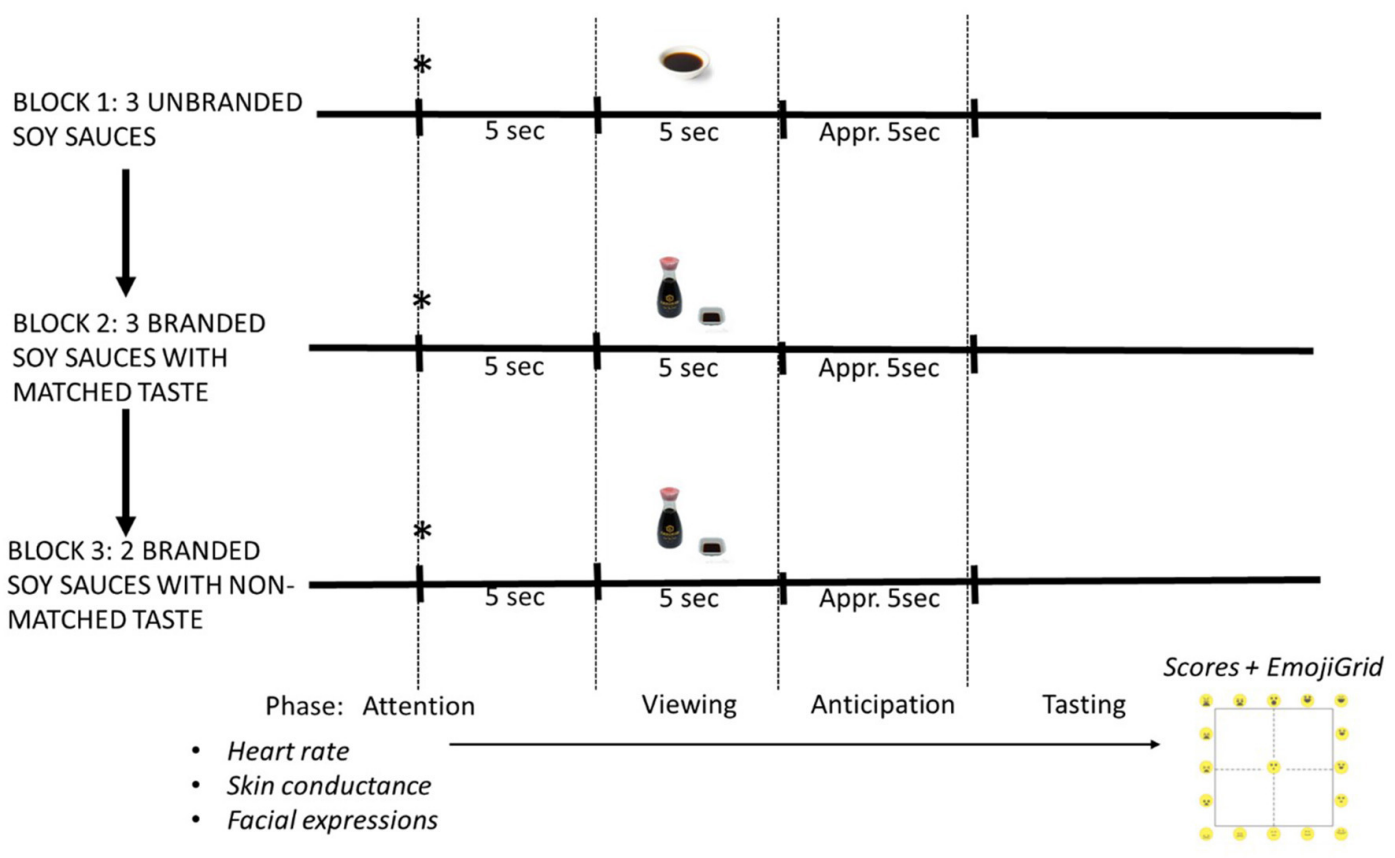
Schematic representation of the study procedure. Participants are presented with three blocks of trials. Each trial starts with an attention signal (asterisk), followed by an image of branded or unbranded image soy sauce, an anticipation period, and a period where rice with soy sauce is chewed. Heart rate, skin conductance and facial expressions are recorded continuously. VAS and EmojiGrid scores are recorded at the end of each trial.

Next, participants received three blocks of stimulus trials in which he/she received taste samples of cooked rice mixed with one of three soy sauces. In each trial, participants, either familiar or unfamiliar with soy sauces, first received an auditory warning signal followed after 5 s by an image (*viewing phase*) for 5 s showing either a generic volume of soy sauce in a white cup (unbranded block 1), or the same white cup with soy sauce combined with the bottle of one of the soy sauces showing the package of the product (blocks 2 and 3, with matched branding and non-matched branding, respectively), followed by an anticipatory waiting period of ~5 s during which no image was shown. Next, the image reappeared and the participants were given a spoon of the mixture of rice and soy sauce in the mouth by the experimenter who simultaneously pressed the enter key of the keyboard to signal the start of tasting (*tasting phase*) in the data recordings. In the branded match block 2, the brand shown in the image and the tasted soy sauce were the same, whereas they were different in the branded non-match block 3. After each sample, which was chewed typically for at least 10 seconds, the participant first scored the liking of the sample using a visual analog scale (VAS) on the screen anchored at the extremes with “dislike it very much” and “like it very much,” followed by a just-right scale for saltines. The just-right scale was anchored at the extremes with “Not salty enough” and “too salty, with “exactly salty enough” in the middle. Next, participants indicated the perceived valence and arousal using a so-called “EmojiGrid” (Toet et al., 2018; Kaneko et al., 2019b, see Figure 1). Only the EmojiGrid arousal scores were analyzed in this study. ANS responses and facial expressions were recorded during each of the phases in all trials. Each of the three soy sauces was presented twice in block 1 and four times in block 2 in a randomized order. To avoid possible unwanted interference of the non-matched condition with the other conditions, the non-matched condition was tested last in block 3 with only two of the three soy sauces (Kikkoman and Formula 1) presented once. This resulted in a total number of 20 trials presented at a rate of ~1 trial per 150 s. The order of the blocks was the same for all participants. The procedures are shown schematically in Scheme 1.

### Physiological ANS Measures

Skin conductance level (or EDA) and heart rates were collected using a BIOPAC MP150 system and AcqKnowledge 5.0.4 data acquisition software (BIOPAC Systems Inc., Santa Barbara, CA). Skin conductance signals were recorded using the exosomatic direct current constant voltage method *via* two pre-gelled, isotonic Ag/AgCl electrodes attached to the thenar and hypothenar eminence of the non-dominant hand. The electrodes were connected through a lead to the photoplethysmogram (PPG)/EDA transmitter that wirelessly sent the signal to the Bionomadix PPGED-R receiver. Heart rate was measured using the TSD200 PPG transducer attached to the index finger of the non-dominant hand. The TSD200 transducer was also connected to the PPG/EDA transmitter. Data was stored on a desktop PC (sample rate 250 Hz).

The PPG signal was processed real-time in AcqKnowledge using the Pulse Rate calculation channel, resulting in the pulse/heart rate in beats-per-minute (bpm).

### Facial Expressions

Video segments of the consumption of each bite were stored together with the participant's code, the product code, and time and date information. Facial expression data were automatically analyzed per time frame of 0.04 s by FaceReader 8.0 (Noldus Information Technology, Wageningen, The Netherlands) in three steps. The face is detected in the first step using the Viola-Jones algorithm (Viola and Jones, 2001). Next, the face is accurately modeled using an algorithmic approach (Den Uyl and Van Kuilenburg, 2008). Based on the Active Appearance method described by Cootes et al. (2001) the model is trained with a database of annotated images that describes over 500 key points in the face and the facial texture of the face. Finally, the actual classification of the facial expressions is based on an artificial neural network trained with 10,000 manually annotated images. The face classification provides the output of seven basic expressions (happy, sad, angry, surprised, scared, disgusted, and contemptuous), one neutral state on the basis of the Facial Action Coding System developed by Ekman and Friesen (1978), three “affective attitudes” (interest, boredom, and confusion), and arousal and valence dimensions based on combinations of facial expressions. Valence scores were calculated per time frame from the FaceReader happiness score minus the most intense FaceReader negative emotion score (sad, angry, surprised, scared, disgusted, and contemptuous). FaceReader scores for each emotional expression, except arousal, range from 0 (emotion is not detected) to 1 (maximal detection) and are based on intensity judgments of human experts. Arousal scores range from−1 to 1. FaceReader allows for the simultaneous presence of multiple emotions. Only the arousal and valence dimensions were used for this study.

FaceReader was validated by others using the Radboud Faces Database, a standardized test with images of expressions associated with basic emotions. The test persons in the images have been trained to pose a particular emotion and the images have been labeled accordingly by the researchers. Subsequently, the images have been analyzed in FaceReader. Accuracy of the assessment of the emotions by FaceReader varied between 84.4% for scared and 95.9 % for happy, with an average of 90% (Bijlstra and Dotsch, 2011). Yet other validation studies showed superior performance of FaceReader for neutral faces (90% correct recognition for FaceReader vs. 59% for humans) (Lewinski, 2015). Another study related FaceReader performance to EMG activity of specific facial muscles that have been associated with specific emotions, namely the zygomaticus major or cheek muscle (for happy emotions) and corrugator supercilii or brow muscle (for angry emotions). Indeed, FaceReader assessment of happy expressions was significantly correlated (*r* = 0.72, *p* < 0.001) with zygomaticus activity, and assessment of angry expressions (*r* = 0.51, *p* < 0.05) with corrugator activity.

A more detailed description of the science behind FaceReader can be found at: http://info.noldus.com/free-white-paper-on-facereader-methodology/. Whether all possible emotional expressions can be categorized by these six emotions, affective attitudes and arousal/valence dimensions remains a matter of debate (e.g., Scherer, 2005).

### Data Analysis

#### Physiological Responses and Facial Expressions

Heart rate frequencies were not successfully collected for five participants. Results were visually inspected and per trial and phase (viewing and tasting phase) parameters were determined as change relative to the baseline value at the beginning of the phase. Heart rates can show for the first couple of seconds an initial decrease in heart rate associated with for example an orienting response, followed by an increase. Parameters for heart rate were: HR min (maximum decrease in heart rate in delta beats per min (delta BPM) during the first 3–4 s), T min (time at HR min), HRmax (maximum heart rate increase during the five seconds of the viewing phase and during the tasting phase, which typically lasted 10–15 s), and TMax (time in seconds at HRmax). The parameter for skin conductance level (SCL) was SCLMax (maximum skin conductance deviation in μSiemens during 5 s for the viewing phase and during the tasting phase). The parameters for facial expressions associated with arousal and valence were FR max arousal, the maximum increase in arousal relative to T0 for 5 s, and FR min valence, the maximum decrease in facial expressions associated with valence relative to T = 0 for 5 s.

#### Scores on Liking, Saltiness, and EmojiGrid Scores

The positions of the scores on the VAS and the EmojiGrid arousal axis were converted into numerical values (1–10).

ANS response and facial expressions were only analyzed for the viewing and tasting phases, not for the anticipation phases. The anticipation phase was less well-controlled with regard to the timing (could vary between 5 and 8 s), and the results were not critical for the experimental questions of this study. The other results were analyzed with Mixed Model Anovas (IBM® SPSS® statistics, version 25, Armonk, New York, USA) with the participant as a random factor, and with the usage of soy sauce (high- and low-frequency user), soy sauce, and condition as fixed factors. Effects of matched branding conditions were tested against unbranded conditions for three soy sauces. Effects of non-matched branding conditions were tested against match branding conditions for the two soy sauces. The usage of soy sauce was a between-subject factor, and the others were within-subject factors. LSD *post-hoc* tests were used for main effects. Ninety-five percent Confidence intervals were used for *post-hoc* tests of interaction effects. Estimated marginal means will be reported in the results and in Table 1. Note that the means reported in the results and in the table may deviate somewhat due to the different models used for their estimation.

**Table 1A:** Estimated marginal means of changes relative to baseline in heart rate, facial expressions, and skin conductance (SCL) during viewing and tasting of soy sauces by low- and high frequency users.

		**Viewing**											
	**Condition**	**Unbranded**						**Matched branded**					
	**Usage frequency**	**High**			**Low**			**High**			**Low**		
	**Product**	**Formula 1**	**Kikkoman**	**Inproba**	**Formula 1**	**Kikkoman**	**Inproba**	**Formula 1**	**Kikkoman**	**Inproba**	**Formula 1**	**Kikkoman**	**Inproba**
	SCL Max	−0.009^bcd^	0.127^a^	0.027^abc^	−0.106^d^	0.020^abc^	0.002^bc^	−0.053^bcd^	−0.080^cd^	−0.056^bcd^	0.032^ab^	−0.007^bcd^	−0.063^bcd^
Fac. Expr.	FR Arousal	0.037^ef^	0.024^f^	0.036^f^	0.042^def^	0.055^cde^	0.037^ef^	0.063^bcd^	0.074^abc^	0.072^bc^	0.093^a^	0.078^ab^	0.080^ab^
	FR Valence	−0.144^ab^	−0.161^abc^	−0.151^abc^	−0.137^a^	−0.147^ab^	−0.147^ab^	−0.178^abcd^	−0.201^cd^	−0.192^bcd^	−0.202^cd^	−0.186^abcd^	−0.229^d^
Heart rate	T MIn	1.090^efg^	0.767^g^	1.043^fg^	0.840^g^	0.690^g^	1.330^def^	1.568^cde^	2.490^a^	2.021^abc^	1.753^bcd^	2.102^ab^	2.400^a^
	HR Min	−1.979^a^	−1.202^a^	−2.107^a^	−2.764^ab^	−1.729^a^	−3.919^b^	−1.738^a^	−1.748^a^	−2.284^ab^	−1.538^a^	−1.356^a^	−1.983^a^
	T max	2.754^def^	2.615^ef^	3.171^cde^	2.840^cde^	2.570^ef^	3.320^c^	3.184^cde^	4.108^ab^	2.200^f^	3.588^bc^	4.290^a^	3.177^cd^
	HR Max	4.019^ab^	4.242^ab^	3.586^ab^	2.115^b^	4.165^a^	−0.230^c^	3.861^ab^	2.473^ab^	4.253^ab^	2.899^ab^	2.922^ab^	3.213^ab^

**Table 1B:** 

		**Tasting**											
	**Condition**	**Unbranded**						**Matched branded**					
	**Usage frequency**	**High**			**Low**			**High**			**Low**		
	**Product**	**Formula 1**	**Kikkoman**	**Inproba**	**Formula 1**	**Kikkoman**	**Inproba**	**Formula 1**	**Kikkoman**	**Inproba**	**Formula 1**	**Kikkoman**	**Inproba**
	SCL Max	−0.135^ab^	−0.198^ab^	−0.228^b^	−0.168^ab^	−0.161^ab^	−0.157^ab^	−0.096^a^	−0.099^a^	−0.102^a^	−0.111^a^	−0.193^ab^	−0.148^ab^
Fac. Expr.	FR Arousal	0.036^e^	0.046^cde^	0.044^de^	0.054^cde^	0.033^e^	0.043^de^	0.079^ab^	0.052^cde^	0.065^abcd^	0.069^abc^	0.082^a^	0.064^abcd^
	FR Valence	−0.226^abc^	−0.235^abc^	−0.220^ab^	−0.213^a^	−0.202^a^	−0.211^a^	−0.285^bcd^	−0.336^d^	−0.290^cd^	−0.309^d^	−0.294^cd^	−0.329^d^
Heart rate	T MIn	3.033^a^	3.011^a^	3.022^a^	1.888^c^	1.838^c^	2.368^abc^	3.067^a^	3.169^a^	2.217^bc^	1.836^c^	1.783^c^	2.917^ab^
	HR Min	−0.963^a^	−0.679^a^	−0.587^a^	−0.574^a^	−0.075^a^	−0.464^a^	−0.550^a^	0.620^a^	−0.380^a^	−0.300^a^	−1.287^a^	0.331^a^
	T max	8.299^bcd^	8.081^cd^	9.785^abc^	9.443^abcd^	8.238^cd^	10.348^a^	10.765^a^	9.184^abcd^	8.419^bcd^	8.847^abcd^	8.228^cd^	10.255^a^
	HR Max	7.026^c^	8.197^bc^	7.667^bc^	9.499^ab^	11.214^a^	10.995^a^	7.870^bc^	9.697^ab^	7.866^bc^	8.642^bc^	8.574^bc^	8.762^bc^

**Table 1C:** 

		**Tasting**							
	**Condition**	**Matched branded**				**Non-matched branded**			
	**Usage frequency**	**High**		**Low**		**High**		**Low**	
	**Product**	**Formula 1**	**Kikkoman**	**Formula 1**	**Kikkoman**	**Formula 1**	**Kikkoman**	**Formula 1**	**Kikkoman**
	SCL Max	−0.095^a^	−0.098^a^	−0.111^ab^	−0.192^ab^	−0.090^a^	0.003268^b^	−0.130^ab^	−0.092^a^
Fac. Expr.	FR Arousal	0.078^c^	0.051^b^	0.068^c^	0.082^c^	0.042^ab^	0.056^b^	0.051^b^	0.023^a^
	FR Valence	−0.285^c^	−0.331^c^	−0.308^c^	−0.293^c^	−0.272^c^	−0.244^bc^	−0.133^a^	−0.192^ab^
Heart rate	T MIn	2.675^a^	3.875^a^	3.354^a^	2.943^a^	3.067^a^	3.169^a^	3.033^a^	3.011^a^
	HR Min	−0.411^a^	0.615^a^	−1.004^a^	−0.649^a^	−0.593^a^	0.520^a^	0.156^a^	−0.982^a^
	T max	9.992^a^	10.154^a^	8.094^b^	8.708^ab^	10.743^a^	9.154^b^	8.143^b^	7.849^b^
	HR Max	8.270^a^	9.723^a^	7.059^a^	8.218^a^	12.698^b^	13.478^b^	11.877^b^	10.214^ab^

*Parts A and B of the table shows the values in the unbranded and match branded conditions during viewing (A) and tasting (B). The lower part C shows the values during tasting of the non-matched branded condition and the corresponding matched-branded condition. Values that share the same character are not significantly different*.

## Results

### General

**Figure 2:**
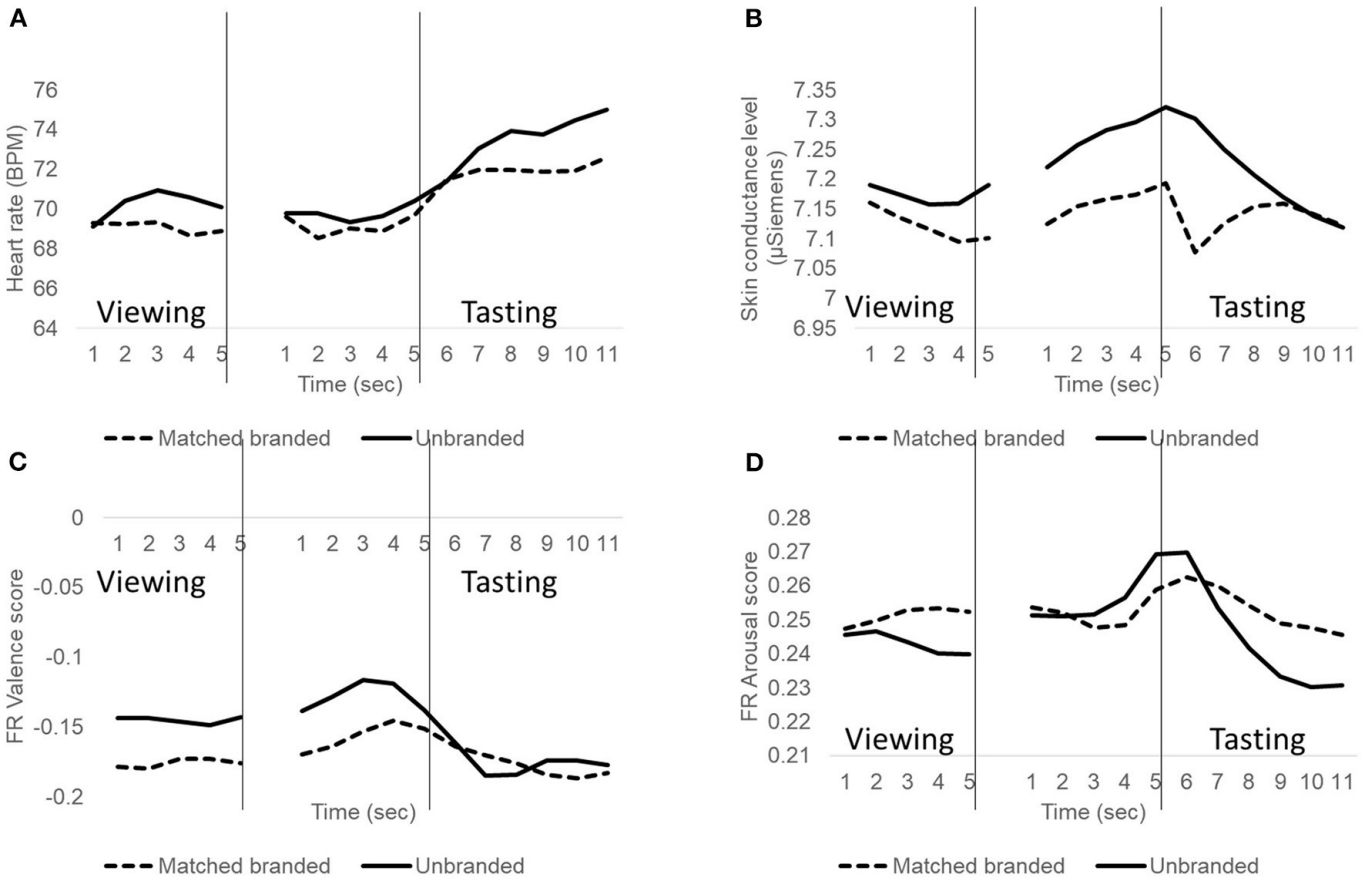
Heart rate **(A)**, skin conductance level **(B)**, and facial expressions related to valence **(C)** and arousal **(D)** during viewing, and tasting of unbranded (solid line) and matched branded (dashed line) stimuli. Viewing and tasting start at time = 1 s. Results of the 5 s preceding tasting are also included. Results are absolute values and were averaged across participants and soy sauces.

Absolute heart rates were stable or decreasing during viewing and anticipation and increasing during the tasting. Absolute skin conductance levels, indicative for arousal, decreased during viewing, increased during anticipation, and again decreased during the tasting. Absolute arousal scores of facial expressions showed a similar pattern. Facial expressions related to valence were stable during viewing, gradually increased during anticipation, and decreased during the tasting. Systematic differences were observed between unbranded and branded conditions during viewing and tasting (see Figure 2). These differences will be explored further in the analysis of the relative changes compared to baseline. Averaged relative changes as well as their significance are shown in Table 1.

Results of ANOVAs on the effects of soy sauce, usage, and branding during viewing and tasting will be described next.

### Implicit Valence and Arousal During Viewing

Viewing the soy sauce bottle with soy sauce in the matched branding condition increased Tmin from 0.96 to 2.06 s [*F*_(1, 150)_ = 72.1, *p* < 0.001] and Tmax from 2.87 to 3.42 s [*F*_(1, 151)_ = 11.2, *p* = 0.001]. Branding did not affect HRmin but did affect HRmax where the effects varied with branding condition and soy sauce [interaction branding condition × soy sauce: *F*_(2,149)_ = 3.5, *p* = 0.03]. With branding, HR max was lowest (+2.7 BPM) for Kikkoman and highest for Inproba (+3.7 BPM). Unexpectedly, soy sauce effects were also found for the unbranded condition, where unbranded soy sauces were presented. The product effects in the unbranded and branded conditions were not consistent: in the unbranded condition HRmax was lowest for Inproba (+1.7 BPM) whereas it was highest in the branded condition. Heart rate parameters did not vary between high- and low frequency users.

Viewing the soy sauce bottle in the matched branded condition decreased arousal measured with skin conductance level for high frequency users high-frequency users from 0.05 to −0.06 μSiemens whereas skin conductance level was unchanged for low frequency users [interaction *F*_(1, 190)_ = 4.1, *p* = 0.05] In contrast, viewing of the soy sauce bottle increased arousal measured with facial expressions [*F*_(1, 189)_ = 53.1, *p* < 0.001]. Viewing of the soy sauce bottle reduced valence measured with facial expressions significantly [*F*_(1, 186)_ = 16.7, *p* < 0.001]. These effects of branding on facial expressions did not vary with usage and/or soy sauces.

### Explicit Liking and Arousal During Tasting

Taste liking and arousal scores were not affected by branding condition. Liking scores varied significantly between soy sauces [*F*_(2,190)_ = 6.8, *p* = 0.001]. The familiar Kikkoman soy sauce was liked significantly better (5.91, *p* < 0.01) than the unfamiliar Formula 1 soy sauce (5.44) and familiar Inproba soy sauce (5.00). In addition, high- and low- frequency users varied in their liking for the various soy sauces, as indicated by the significant interaction between product and usage [*F*_(2,190)_ = 3.7, *p* = 0.03]. *Post-hoc* tests showed that low-frequency users liked Formula 1 (5.81 vs. 4.98, *p* < 0.01) and Inproba (5.46 vs. 4.60, *p* < 0.01) significantly better than high-frequency users, whereas Kikkoman were liked as much by both usage groups (5.74 vs. 5.92, n.s.).

Emoji arousal also varied between soy sauces [*F*_(2,190)_ = 4.5, *p* = 0.01] with significantly higher arousal for Kikkoman (3.23) and Formula 1 (3.21) compared to Inproba (2.71).

### Implicit Valence and Arousal During Tasting

First the results for the unbranded and matched branded conditions will be presented, followed by the results of the matched branded and non-matched branded conditions. HRmin and Tmin during tasting did not vary with branding condition, product and/or usage group. During the tasting, HRmax decreased after matched branding from 9.6 to 8.1 BPM [*F*_(1, 136)_ = 7.8, *p* = 0.006]. The effects of matched branding varied with the usage group [interaction *F*_(1, 136)_ = 6.1, *p* = 0.02]. In fact, the reduction in BPM after branding was primarily caused by the low-frequency users (10.6–8.7 BPM, *p* < 0.05), whereas HR max was unaffected for the high-frequency users (8.7 and 8.5 BPM, n.s.).

Matched branding significantly lowered the valence of facial expressions from−0.22 to−0.31 [*F*_(1,188)_ = 37.3, *p* < 0.001], irrespective of usage and soy sauces.

Arousal (SCLmax) decreased gradually during the tasting, but the decrease was significantly smaller after matched branding [−0.125 vs. −0.174 μSiemens, *F*_(1,190)_ = 5.4, *p* = 0.02], indicating higher arousal after matched branding. This result was corroborated by the results of facial expressions that showed significantly higher arousal after branding [0.068 vs. 0.043, *F*_(1, 187)_ = 19.4, *p* < 0.001]. In addition, these effects also varied with soy sauce and usage [*F*_(2,187)_ = 3.2, *p* = 0.04]. *Post-hoc* testing showed no significant differences in facial expressions of arousal in the unbranded condition between products and usage groups (average FR score was 0.043). In the matched branded condition, the facial expressions of arousal increased to 0.082 for Kikkoman for the Low-frequency users and to 0.079 for Formula 1 for the high-frequency users.

Compared to matched branding, HR max increased significantly [+12.1 vs. +8.3 BPM, *F*_(1, 65)_ = 27.3, *p* < 0.001] with non-matching branding information, irrespective of product or usage group. Non-matching branding information also increased SCL max significantly [*F*_(1, 111)_ = 5.4, *p* = 0.02]. This increase was almost exclusively caused by Kikkoman soy sauce and high-frequency users (−0.09 to +0.15 μSiemens) [interaction *F*_(1, 105)_ = 6.3, *p* = 0.01]. In contrast, arousal with facial expressions decreased from 0.07 to 0.04 with non-matching information [*F*_(1, 111)_ = 12.5, *p* = 0.001]. This effect varied with soy sauce and usage [3-way interaction *F*_(1, 108)_ = 7.5, *p* = 0.007]. This interaction was primarily caused by Kikkoman for low-frequency users where arousal with facial expressions decreased from 0.082 with matching branding to 0.023 with non-matching branding.

## Discussion

This study compared the results of traditional sensory tests (explicit subjective responses), physiological tests of ANS activity, and behavioral expressive (facial expression) tests to (1) variations in consumers' familiarity with soy sauces, (2) variations in intrinsic food properties by comparing responses to the test soy sauces, and (3) variations in expectations by comparing responses to unbranded soy sauces to branded (matched and non-matched) responses.

The results confirmed the hypothesis formulated in the introduction. The effects of usage of soy sauce on skin conductance during viewing provide evidence for the first hypothesis: expectations will vary with the frequency of soy sauce use. The systematic effects of brand of soy sauce on heart rate parameters during viewing, supports the second hypothesis [different brand information (product) will produce different expectations]. The fact that branding affects heart rate, skin conductance and facial expressions during viewing as well as tasting supports the third hypothesis: expectations affect responses to the brand name and to the taste stimulus. The specific relationship between viewing and tasting responses is not always clear: when the taste confirmed the branding information in the matched branding condition, the heart rate during tasting is reduced compared to the unbranded condition, which supports Mandler's incongruity theory. However, skin conductance increases, which does not support Mandler's theory. The relatively strong effects on heart rate and skin conductance during tasting when the taste disconfirmed the branding information (non-matched branding condition) again supports Mandler's theory. Further branding studies in the food domain are clearly needed to come to firm conclusions regarding the underlying mechanisms. Further studies may also investigate the possibility that some of the branding results found in this study are related to the fact that unbranded conditions were tested first, followed by match-branded and non-matched branded conditions. This means that participants were already somewhat familiar with the taste of the soy sauces and with the general procedure when the branded trials started. The reason for selecting this order was to avoid (1) that brand information interfered with unbranded taste responses, and (2) that non-matched branding interfered with matched branding.

The results showed that explicit liking and arousal scores were primarily affected by the taste of the specific soy sauce and by the participants' previous experience with soy sauces. These subjective scores were not affected when matching branding information was provided or when this branding information was incorrect. These results are in line with those from a previous study where liking scores also varied between brands of soy sauces, and no overall effects of branding on liking scores were found (Ushiama et al., 2021). In the present study, relative changes in facial expressions related to valence and arousal, physiological skin conductance levels, and heart rates showed relatively small effects of the taste of the specific soy sauces (except for in the non-matched branding condition). Instead, these measures were primarily affected during tasting by (1) whether branding information was provided, (2) whether the branding information matched the taste, and (3) the participants' level of experience with soy sauce.

These results suggest that explicit scores reflect primarily the food's intrinsic taste and flavor properties and that they are less sensitive to other factors that determine the food experience, such as expectations based on branding information. As shown by the absolute values in Figure 1, branding information increases heart rate, skin conductance levels and affects facial expressions, especially during the viewing and anticipation phases. Typically, heart rates, facial expressions and skin conductance levels are relatively stable during viewing, increase during anticipation and decrease during tasting (except for heart rate for which the continued increase during tasting is probably related to the muscle activity during chewing). Frequent users, i.e., users who are highly familiar with the tastes and brand names of soy sauces, probably form stronger expectations based on branding information, resulting in stronger anticipation responses. During the subsequent tasting, physiological measures, and facial expressions continued to be affected by these expectations based on branding or usage, especially when the expectation based on branding proved to be wrong in the non-matching condition.

The sensitivity of physiological measures and facial expressions to non-matching branding information may reflect an important function of the autonomic nervous and expressive systems, to monitor the environment with regard to sudden events that may require approach or avoidance reactions. As long as the taste matches the expectation based on the branding information, physiological reactions, and facial expressions to the taste are relatively mild. However, if taste expectations are not met in the non-matched condition, skin conductance level and heart rate increase, possibly in the preparation of an avoidance reaction. Obviously, replacing the taste of one soy sauce with the taste of another soy sauce is a rather innocent manipulation that offers no real threat to the consumer's safety, and consequently, no avoidance reaction such as spitting out the food is required. The fact, however, that a reaction was measured at all demonstrates the sensitivity of the system for changes in the environment, even when these changes are small and harmless. Future studies may compare discrimination performance based on implicit responses to the more traditional explicit discrimination tests. Sensitivity of physiological measures may also explain the unexpected finding of an effect of soy sauce brand on heart rate during viewing of unbranded soy sauces. This effect had also been observed previously in other studies from our laboratory. Possibly, these effects are caused by carry-over effects of preceding trials, i.e., the taste of a stimulus on one trail may affect the responses on the next trial, even when inter-trial times are sufficiently long.

Interestingly, physiological responses and facial expressions not only reacted to the non-match of expectations and taste in general but varied with the soy sauce brand. This brand-specificity in physiological responses and facial expressions was found to a lesser degree, and only for heart rate, during expectations and tasting in matching conditions. This result demonstrates that the ANS system is sensitive enough to detect relatively small differences in taste experiences, but that this happens primarily in situations where expectations and taste experiences do not match. The reason for the brand-specificity during the non-matched tasting is not clear. If the responses would reflect only the discrepancy between expectations and actual taste, then the effects should be similar for the situations where the taste of brand B follows the image of brand A and vice versa.

The higher sensitivity of physiological reactions and facial expressions compared to explicit scores for factors such as branding that are not directly related to the intrinsic food properties, is in line with our previous study. In that study, participants evaluated foods repeatedly in their own and in the sensory laboratory (De Wijk et al., 2019). The test location was the only factor that was varied. All other factors, such as time of the day, day of the week, and sample preparation and presentation procedure, were kept constant. Similar to the present study, the results showed that explicit scores reflected primarily the differences between the test foods, whereas the physiological heart rate and facial expressions reflected differences between test locations as well. Combined, the results of these studies suggest that physiological measures and facial expressions reflect experiences in general, and not only experiences directly related to foods. These experiences may be related to a specific mood that is affected by the food that is consumed and the circumstances in which the food is consumed. These circumstances involve the physical and social food environment, but also whether one is hungry or not.

Limitations of this study are the relatively small number of participants, especially when subgroups based on familiarity are used, and the small number of replications (especially in the non-match condition). Furthermore, the fact that only one specific type of food (condiment) was investigated makes generalization to other foods difficult.

In conclusion, this study suggests that liking scores may be most sensitive to the food's intrinsic taste and texture properties, whereas implicit measures and facial expressions may be most sensitive to extrinsic properties such as brand name and packaging. Both implicit and explicit measures were affected by the consumers' previous food experiences.

## Data Availability Statement

The raw data supporting the conclusions of this article will be made available by the authors, without undue reservation.

## Ethics Statement

The studies involving human participants were reviewed and approved by Social Ethics Committee of Wageningen University and Research. The patients/participants provided their written informed consent to participate in this study.

## Author Contributions

SU, MV, DK, and RW conceived the original idea for this study and designed the study. SU, PZ, and MU set-up and conducted the study. RW analyzed the data and took the lead in writing the manuscript. All authors provided critical feedback and helped shape the research, analysis, and manuscript.

## Conflict of Interest

SU and DK were employees of Kikkoman Europe R&D Laboratory B.V., during the conduct of the study. A possible conflict of interest was prevented by following the WUR-integrity code (https://www.wur.nl/en/About-Wageningen/Integrity-and-privacy/Scientific-integrity.htm). PZ was employed by Noldus Information Technology. The remaining authors declare that the research was conducted in the absence of any commercial or financial relationships that could be construed as a potential conflict of interest.
